# Assessment of the retinal posterior pole in dominant optic atrophy by spectral-domain optical coherence tomography and microperimetry

**DOI:** 10.1371/journal.pone.0174560

**Published:** 2017-03-30

**Authors:** Massimo Cesareo, Elena Ciuffoletti, Alessio Martucci, Jacopo Sebastiani, Roberto Pietro Sorge, Eleonora Lamantea, Barbara Garavaglia, Federico Ricci, Andrea Cusumano, Carlo Nucci, Francesco Brancati

**Affiliations:** 1 Ophthalmology Unit, Department of Experimental Medicine and Surgery, University of Rome “Tor Vergata”, Italy; 2 Laboratory of Biometry, Department of Systems Medicine, University of Rome “Tor Vergata”, Rome, Italy; 3 Molecular Neurogenetics Unit, Neurological Institute C. Besta IRCCS, Milan, Italy; 4 Department of Life, Health and Environmental Sciences, University of L'Aquila, L'Aquila, Italy; 5 Laboratory of Molecular and Cell Biology, Istituto Dermopatico dell’Immacolata IRCCS, Rome, Italy; Bascom Palmer Eye Institute, UNITED STATES

## Abstract

**Background:**

To assess posterior pole (PP) retinal structure in patients with genetically confirmed autosomal dominant optic atrophy (ADOA) using new spectral domain optical coherence tomography (SD-OCT) segmentation technology. To analyze retinal PP thickness in relation to retinal sensitivity data from microperimetry (MP) in ADOA patients.

**Methods and findings:**

This prospective cross-sectional study included 11 patients with ADOA and 11 age-matched healthy subjects. All participants underwent both a “Posterior Pole” and “peripapillary RNFL (pRNFL)” scanning protocol using SD-OCT. Functional mapping of the PP was also performed using MP. A customized program was implemented in order to achieve accurate superimposition of MP sensitivity map onto SD-OCT map. The thickness of the PP different retinal layers and pRNFL was obtained and measured for each eye. Mean retinal sensitivity values and fixation stability were obtained and compared between ADOA patients and healthy subjects. Correlation analysis was performed on a point-to-point basis to evaluate the association between mean thickness and retinal sensitivity of each retinal layer. Total retinal thickness (TRT), Retinal Nerve Fiber Layer (RNFL), Ganglion Cell Layer (GCL), Inner Plexiform Layer (IPL), Inner Nuclear Layer (INL) and Inner Retinal Layers (IRL) at the posterior pole as well as pRNFL were significantly thinner in ADOA patients (*P* < 0.0001). On the contrary, the Outer Plexiform Layer (OPL) and the Outer Nuclear Layer (ONL) were significantly thicker in the ADOA group (*P* < 0.001). No significant differences were found in Retinal Pigment Epithelium (RPE) and Outer Retinal Layers (ORL) thickness between ADOA and controls. The average PP retinal sensitivity was significantly reduced in ADOA patients compared with controls (*P* < 0.001), as measured by microperimeter Nidek MP-1 (MP1). Fixation stability was significantly worse in the ADOA group (*P* = 0.01). The most severe sensitivity defects in ADOA patients were found at the level of the papillo-macular bundle (PMB).

**Conclusions:**

Inner retinal layers showed pathological changes in ADOA patients. In addition, the whole retinal PP (not only the PMB) was significantly altered in ADOA, both in terms of retinal thickness and sensitivity.

## Introduction

Autosomal Dominant Optic Atrophy (ADOA, or Optic Atrophy 1; OMIM#165500), also known as Kjer disease, is the most common form of hereditary optic neuropathy [1] with an estimated incidence of 1/30.000 people worldwide [2].

ADOA, generally diagnosed in early childhood, is characterized by a progressive bilateral loss of visual acuity, blue-yellow dyschromatopsia, variable central or centrocecal visual field defects, and temporal or diffuse optic nerve pallor with optic disc excavation [3–5]. Patients with ADOA present with considerably variable clinical features, even within the same family, ranging from subclinical manifestations to legal blindness: this great heterogeneity is indicative of incomplete penetrance [4–6].

Mutations in the optic atrophy-1 gene (*OPA1*, OMIM*605290), which is localized on the long arm of chromosome 3q28-q29, are responsible for about 60%-80% of the cases of ADOA [7–9]. To date, more than 300 *OPA1* mutations have been reported with mutational hot spots in the catalytic GTPase domain (exons 8–15) and the dynamin central domain (exons 16–23) [8–11].

*OPA1* gene codes for a 960-amino-acid, dynamin-related GTPase targeted to the inner mitochondrial membrane, which is involved in multiple functions. *OPA1* plays a major role in regulating mitochondrial network dynamics: in particular, the Opa1 protein induces fusion of the mitochondrial inner membrane, modulates apoptosis through the compartmentalization of cytochrome c and it is also implicated in oxidative phosphorylation and in the maintenance of the membrane potential [12–14].

The gene is ubiquitous, but most strongly expressed in the retina and in the brain [7–8]. Postmortem histopathology studies in ADOA patients reported a selective deficit of the retinal ganglion cell (RGC) layer and the retinal nerve fiber layer (RNFL), with ascending optic nerve atrophy [5,7].

It was thus suggested that the RGCs degenerate first, with optic atrophy developing secondarily [15].

Optical coherence tomography (OCT) is a noninvasive technique that has been successfully used to diagnose and monitor different optic neuropathies, such as glaucoma, Leber Hereditary Optic Neuropathy and Non-Arteritic Ischemic Optic Neuropathy [16–18].

Time-domain OCT (TD-OCT) has been previously used to study patients with ADOA. According to these studies, eyes with ADOA display a significant reduction of the RNFL thickness (RNFLt) in all quadrants, with preferential involvement of the temporal and inferior quadrants; the age-related progression of fiber-layer thinning parallels that seen in healthy controls [19–21].

ADOA is characterized by the early and preferential involvement of the small fibers in the papillo-macular bundle (PMB); this involvement is usually considered as a hallmark of mitochondrial optic neuropathies [22].

Spectral-domain OCT (SD-OCT) has several advantages over TD-OCT, such as increased repeatability and reproducibility and, more recently, the possibility of imaging and quantifying retinal damage by measuring the thickness of each retinal layer [23,24]. Previous studies have evaluated retinal morphology in ADOA patients using SD-OCT [25–28].

Microperimetry (MP) or fundus-perimetry (FP), which enables retinotopic mapping of localised fundus sensitivity and fixation, can be used to find small visual field defects that escape detection with conventional perimetry [29–30].

FP has recently been used to evaluate fixation patterns and macular light sensitivity in ADOA patients [26].

In the present study, we developed and implemented a new experimental customized MP program with the aim of investigating the association between retinal sensitivity measured by MP and retinal structure assessed by SD-OCT.

In particular, SD-OCT was used to evaluate “layer-by-layer” morphology and thickness at the retinal posterior pole (PP) in ADOA patients.

## Materials and methods

All patients included in the present study had a molecular diagnosis of ADOA caused by *OPA1* mutations; they were consecutively enrolled and examined at the Ophthalmological Unit at University of Rome “Tor Vergata” between 2013 and 2015.

All participants gave their written informed consent according to the Declaration of Helsinki; the study was approved by the internal review board of the University Hospital of Tor Vergata, Rome.

11 consecutive patients from 5 unrelated families were recruited.

Exclusion criteria included the presence of any retinal pathology or optic nerve disease other than ADOA, previous intraocular surgery, current use of any drug therapy known to be toxic to the retina and/or optic nerve, and spherical or cylindrical refractive errors higher than 3 and 2 diopters, respectively.

Genetic counseling was offered to all patients and their families to extend testing to other members, interpret genetic results, discuss diagnosis and recurrence risks.

The control group consisted of 11 age- and sex-matched healthy subjects recruited during routine ophthalmologic examinations. Inclusion criteria were as follows: spherical or cylindrical refractive errors lower than 3 and 2 diopters, respectively; normal (<21 mmHg) intraocular pressure; normal appearance of the optic disc; no visual field loss using SITA-Standard program 24–2 and the Glaucoma Hemifield Test of the Humphrey Visual Field Analyzer (model 750, Zeiss Humphrey Systems, Dublin, CA, USA); no significant ocular diseases found by routine ophthalmologic examination; and no family history of glaucoma or systemic diseases with possible ocular involvement such as diabetes mellitus.

Both control group and subjects underwent the same visit protocol.

All subjects had extensive ophthalmologic examinations, including best-corrected visual acuity (BCVA) measurement, Goldmann applanation tonometry, Standard Automated Perimetry (SAP) with SITA-Standard program 24–2 of a Humphrey Field Analyzer, slit-lamp biomicroscopy with dilated fundus examination and fundus digital color retinography.

All participants underwent the “Posterior Pole” scanning protocol using SD-OCT (Spectralis; Heidelberg Engineering, Heidelberg, Germany). Images were acquired using the image alignment eye-tracking software (TruTrack; Heidelberg Engineering GmbH) to obtain volumetric retinal scans comprising 61 single axial scans (scanning area: 30° x 25°) centred on the fovea, with a fovea-to-disc inclination of 7 degrees.

As reported in detail previously [31], Spectralis automated segmentation (software version 6.0) was used to obtain the following thickness measurements: total retinal (retina); retinal nerve fiber layer (RNFL); ganglion cell layer (GCL); inner plexiform layer (IPL); inner nuclear layer (INL); outer plexiform layer (OPL); outer nuclear layer (ONL); retinal pigment epithelium (RPE); inner retinal layers (IRL) and outer retinal layers (ORL).

Peripapillary RNFL (pRNFL) thickness was also assessed in all subjects using the “peripapillary RNFL” scanning protocol of the Spectralis SD-OCT; pRNFL thickness was calculated around the disc with 16 averaged, consecutive circular B-scans (diameter of 3.5 mm, 768 A-scans); an online tracking system was used to compensate for eye movement. pRNFL thickness represents the mean distance between the inner limiting membrane and the posterior boundary of the retinal nerve fiber layer, along a 6° radius circle scan centred on the optic nerve head.

All SD-OCT scans were acquired by the same experienced operator after pupil dilation with 0.5% tropicamide and 10% phenylephrine eye drops (Visumidriatic Fenilefrina, Visufarma).

Only high-quality scans, defined as scans with signal quality > 25, where the quality score range is 0 (poor quality) to 40 (excellent quality), without discontinuity or misalignment, poor illumination, involuntary saccades, or blinking artifacts and absence of algorithm segmentation failure on careful visual inspection were used for analysis. [31]

No manual correction to the Spectralis automatic segmentation of the different retinal layers was necessary.

An internal fixation target was used as this method is reported as having the highest reproducibility [32]. To acquire the PP and the pRNFL scans, the patient was asked to fixate on a central target and on a nasal target, respectively.

Furthermore, all enrolled subjects underwent fundus-related perimetry using Nidek MP-1 (MP1) (software 1.7.3) (Nidek Technologies Srl., Vigonza, PD, Italy). A new microperimetry program, based on the topographical arrangement of the SD-OCT PP protocol, was created and integrated into the library of the MP1 microperimeter program patterns. Threshold strategy was 4–2 with pre-test and a red cross fixation target (3° in diameter and 1° in thickness).

**Fig 1:**
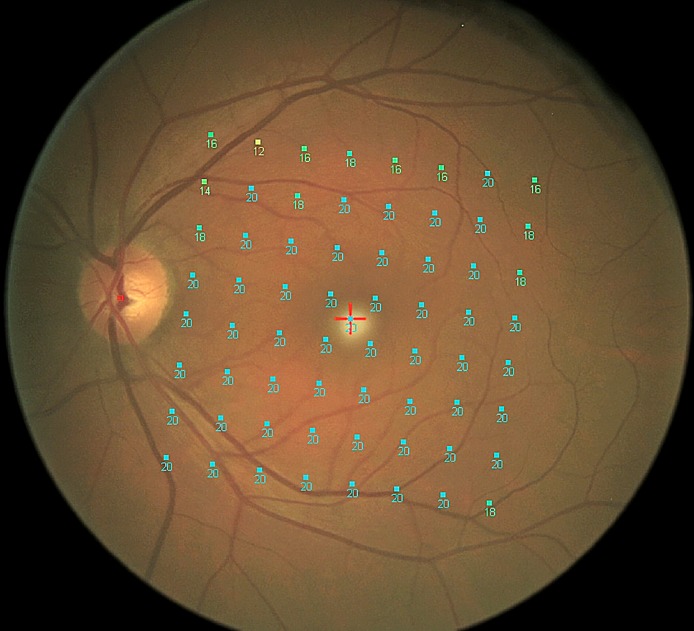
Posterior Pole numeric map of retinal differential light sensitivity of MP1 of a healthy subject.

The new exam pattern of MP1 consists of 64 equidistant test points which take into account the seven degree “fovea to-disc” inclination of the SD-OCT PP protocol (Fig 1), in order to permit easy and accurate overlapping to the Spectralis PP map.

For this reason, spatial coordinates of each test point of the MP program pattern were defined by applying a specific 7° rotation matrix.

In two dimensions, one rotation is a transformation R(θ), that depends on an angle θ, and transforms the vector (x,y) in:

x' = xcosθ − ysinθ
y' = xsinθ + ycosθ

Using the multiplication of the matrix, the rotation can be described as:

$$\left[\begin{array}{c} x'\\ y' \end{array}\right]=\left[\begin{array}{cc} \cos\theta & \text{-}\sin\theta \\ \sin\theta & \cos\theta \end{array}\right]\left[\begin{array}{c} x\\ y \end{array}\right]$$

The square matrix in this expression is an orthonormal matrix of rank two, a transformation known as counterclockwise rotation of angle θ around the origin.

**Fig 2:**
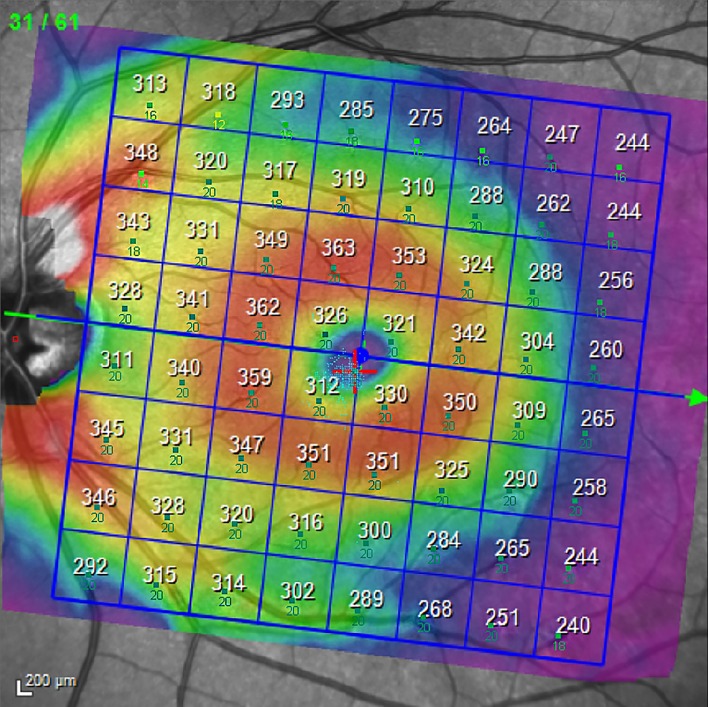
Combination map of SD-OCT PP total retina and MP1 PP map.

The SD-OCT PP map was imported into the MP software; subsequently MP results were superimposed onto the SD-OCT imaging. This “overlapping” produces a combined morpho-functional map of differential light sensitivity values detected by MP1, and PP thickness values provided by the SD-OCT (Fig 2).

Fixation stability was quantified by calculating the bivariate contour ellipse area (BCEA) and the major and minor axis of the ellipses related to the cross fixation target [33,34].

### Statistical analysis

Both eyes of each participant were evaluated, however only one eye was randomly chosen for statistical analysis.

All data were initially entered into an EXCEL database (Microsoft, Redmond, Washington–United States). The analysis was performed using the SPSS vers. 20.0 (SPSS, Chicago, Illinois, USA); the NCSS and PASS vers.11.0 were used for the power analysis.

Descriptive statistics consisted of the mean ± standard deviation values for parameters with Gaussian distributions (after confirmation with histograms and the Kolgomorov-Smirnov test).

The level of statistical power to detect a difference between the two groups was 85% with an alpha = 0.5 for N = 11 subjects.

Comparison among groups was performed by one-way analysis of variance (ANOVA) and by the Bonferroni procedure for multiple tests of significance.

Differential sensitivity values of the 64 test points of the MP1 PP map were calculated on a pointwise basis in ADOA patients and controls.

Also mean sensitivity values as provided by MP1 software using the “polygon tool” and fixation stability by using BCEA were compared between the two groups.

A *P* value of < 0.05 was considered statistically significant.

Linear regressions using coefficient r of Pearson were used to search for correlations between visual acuity and RNFL and other different retinal layers measurements, introducing age and sex as covariates. Pearson’s test was also used to identify statistical correlations between pRNFL and PP RNFL thickness and between perimetric indices (Mean deviation, Pattern Standard Deviation, Visual Field Index) and average retinal thickness.

A ROC curve was calculated in order to identify a cut-off value of layer thickness around which absolute scotomas (0 dB) appear in the microperimetry map.

## Results

11 ADOA patients (mean age: 38± 17.9, range 15–66 years; 5 males and 6 females; mean BCVA: 0.5 logMAR, range 0.0–1.1) from 5 independent families harboring a pathogenic heterozygous mutation in the *OPA1* gene were included in the study.

The demographic, genetic and BCVA data of ADOA affected individuals are presented in Table 1.

**Table 1:** *OPA1* mutations^a^, clinical and demographic data in 9 patients with ADOA.

Family	Patient number	Sex	Age (y)	OPA1 gene site	Nucleotide Change	Amino Acid Change	Visual acuity (R/L)^b^
A	1	F	11	Intron 14	c.1377+3A>T	Splicing defect	0,2/0,1
A	2	M	48	Intron 14	c.1377+3A>T	Splicing defect	0,0/0,1
B	3	M	26	Exon 25	c.2481_2501del	p.Lys828_Lys834del	0,4/0,4
C	4	M	51	Exon 29	c.2873_2876del	p.Val958Glyfs*3	0,7/0,7
C	5	F	43	Exon 29	c.2873_2876del	p.Val958Glyfs*3	0,1/0,1
C	6	F	22	Exon 29	c.2873_2876del	p.Val958Glyfs*3	0,5/0,5
C	7	M	22	Exon 29	c.2873_2876del	p.Val 958Glyfs*3	1,0/0,7
C	8	F	66	Exon 29	c.2873_2876del	p.Val 958Glyfs*3	0,1/0,2
C	9	F	60	Exon 29	c.2873_2876del	p.Val 958Glyfs*3	0,4/0,4
D	10	F	32	Exon 8	c.815T>C	p.L272P	1,2/1,2
E	11	M	22	Exon 19	c.1870delG	Splicing defect	0,5/0,4

^a ^Variants are described according to *OPA1* transcript variant 8 (RefSeq: NM_130837.2)

^b ^R/L: Right/Left Eye

Most mutations were well-established *OPA1* pathogenic mutations listed in the *OPA1* mutation database (www.mitodyn.org).

The control group consisted of 11 age and sex-matched healthy subjects (mean age: 37.7±19.5; range 15–67 years; 5 males and 6 females; mean BCVA: 0.0 logMAR).

Mean refractive error was -1.70 ± 2.31 diopters, ranging from -2.50D to +1.00D.

Signal strength of SD-OCT scans did not differ between the ADOA and healthy groups (*P* = 0.762).

The results of the average thickness of the overall retina and other different retinal layers in ADOA patients and in controls are shown in Table 2.

**Table 2:** Posterior Pole Retinal Layers Thickness values and Statistical Differences between Patients with ADOA and controls.

Group	Thickness (μn)	Retina^a^	RNFL^a^	GCL^a^	IPL^a^	INL^a^	OPL^a^	ONL^a^		RPE^a^		IRL^a^	ORL^a^	
**ADOA**	**Mean**	262,76	22,77	21,45	18,92	30,88	26,40	60,50		13,70		183,80	78,50	
	**SD**^**b**^	24,29	13,61	4,662	4,68	6,52	3,80	12,20		3,20		51,27	3,81	
**Controls**	**Mean**	299,46	42,24	33,61	27,86	32,01	25,70	58,10		13,90		219,97	79,35	
	**SD**^**b**^	34,53	28,15	10,41	9,82	5,27	3,20	10,40		2,02		33,09	5,12	
***P***^**§**^		.0001	.0001	.0001	.0001	.0001	.001		.001	.06	.0001			.34

^**a**^**Retina**: total retinal thickness; **RNFL**: Retinal Nerve Fiber Layer; **GCL**: Ganglion Cell Layer; **IPL**: Inner Plexiform Layer; **INL**: Inner Nuclear Layers; **OPL**: Outer Plexiform Layer; **ONL**: Outer Nuclear Layer; **RPE**: Retinal Pigment Epithelium; **IRL**: Inner Retinal Layers; **ORL**: Outer Retinal Layers

^b^**SD**: Standard Deviation

^c^***P*:** Significant difference (p<0.05) in Anova test between normal and ADOA groups for each population.

**Fig 3:**
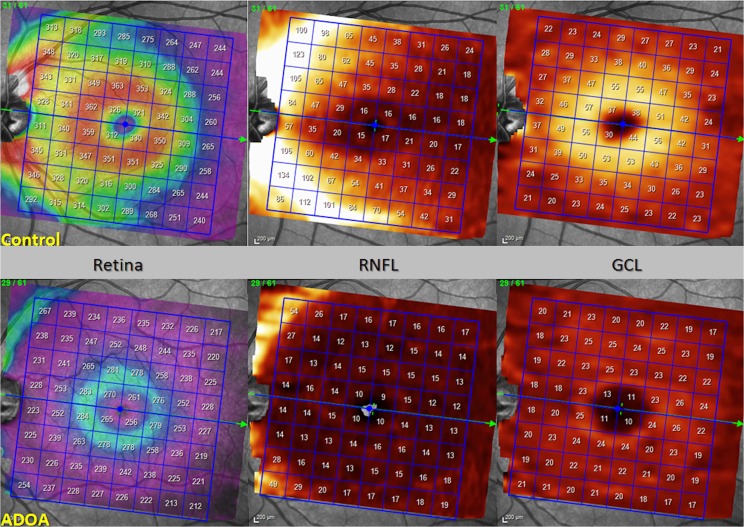
SD-OCT PP map of total retina, RNFL and GCL thickness of a healthy subject vs ADOA patient.

**Fig 4:**
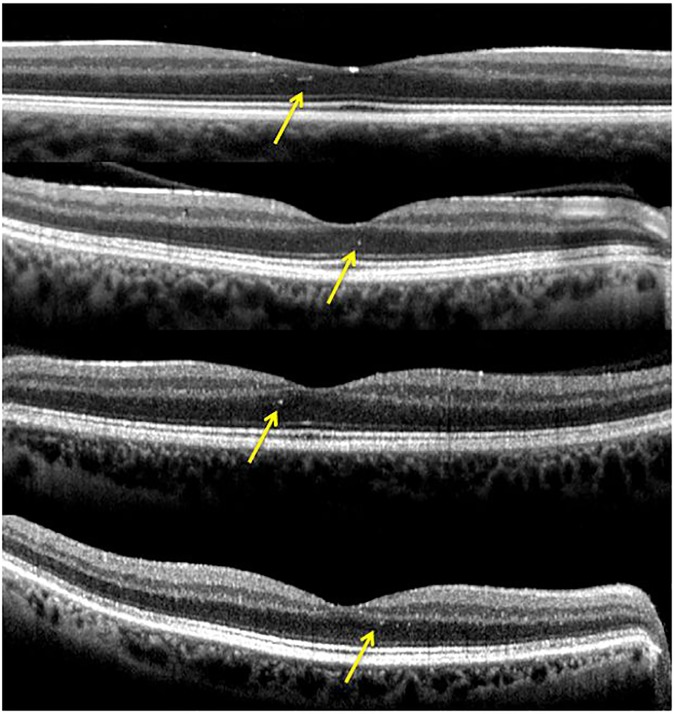
Hyperreflective dots in 4 of 11 ADOA patients at the level of the parafoveal ONL.

Measurements of the retinal layers provided by the new segmentation application of the Spectralis OCT showed reduced thickness of overall retina, RNFL, GCL, IPL, INL and IRL in patients with ADOA (*P* < 0.0001; Fig 3) while OPL and ONL thickness proved to be increased in ADOA patients (*P* < 0.001). Furthermore, the presence of some hyperreflective dots in 4 of 11 (36,4%) ADOA patients at the level of parafoveal ONL was found (Fig 4).

On the contrary, RPE and ORL did not significantly differ between the two groups.

pRNFL thickness measurements provided by SD-OCT device revealed that all RNFL sectors were affected to a greater degree in patients with ADOA than in healthy controls (*P* < 0.001), as shown in Table 3.

**Table 3:** Peripapillary RNFL thickness values provided by RNFL-N Axonal Analytics and Statistical Differences in Autosomal-Dominant Optic Atrophy (ADOA) and Control Subjects.

Group	Thickness (μm)	PMB^a^	TI^a^	T^a^	TS^a^	NI^a^	N^a^	NS^a^	G^a^	N/T^a^
**ADOA**	**Mean**	20,22	92,38	27	67,67	73	55,28	82,5	60,06	2,20
	**SD**^**b**^	6,025	19,01	5,77	18,62	12,99	10,72	18,83	8,46	0,62
**Controls**	**Mean**	51,8	143,2	66,8	137,3	117,1	76,6	107,6	99	1,16
	**SD**^**b**^	5,5	13,0	5,4	19,5	24,3	17,9	28,3	12,4	0,3
***P***^**c**^		.001	.001	.001	.001	.001	.001	.001	.001	.001

^a^**PMB**: papillo-macular bundle; **TI**: temporal-inferior; **T**: temporal; **TS**:temporal-superior; **NI**: nasal-inferior; **N:** nasal; NS: nasal-superior; G: global; N/T: nasal-to-temporal ratio

^b^**SD**: Standard Deviation

^**c**^***P***: Significant difference (*P*<0.05) in Anova test between normal and ADOA groups for each population.

Pearson's correlation coefficient analysis showed that PP RNFL thickness is correlated strongly with pRNFL thickness values (r = 0.798) in ADOA patients.

The average PP retinal sensitivity provided by MP1 was significantly reduced in ADOA patients compared with controls (*P* < 0.001) on a pointwise basis as shown in Table 4.

**Table 4:** Mean ± standard deviation retinal sensitivity values of the 64 test-points measured by the MP1 “posterior pole” program in Autosomal-Dominant Optic Atrophy (ADOA) and Control Subjects.

**ADOA**									
**N**^**a**^	**S**^**a**^								**T**^**a**^
	11,45 ± 3,44	12,23 ± 3,07	14,18 ± 3,11	15,23 ± 2,32	14,18 ± 2,95	14,41 ± 1,65	13,77 ± 2,63	12,64 ± 4,46	
	12,64 ± 4,16	14,05 ± 3,69	14,82 ± 3,67	16,23 ± 3,61	15,32 ± 4,45	15,50 ± 3,73	14,86 ± 3,28	14,68 ± 3,10	
	13,18 ± 4,15	14,68 ± 4,47	15,50 ± 5, 44	15,41 ± 6,13	16,82 ± 2,42	17,41 ± 3,94	15,64 ± 4,35	15,05 ± 4,20	
	12,27 ± 4,69	14,32 ± 5,82	15,41 ± 4,95	14,86 ± 5,31	15,41 ± 5,22	17,41 ± 4,81	16,64 ± 3,30	16,41 ± 2,26	
	6,22 ± 6,69	11,32 ± 6,29	12,28 ±6,78	11,23 ± 8,92	16,64 ± 4,81	16,91 ± 4,23	17,36 ± 3,09	16,0 ± 3,13	
	9,40 ± 6,63	9,54 ± 7,20	11,5 ± 7,20	12,50 ± 7,54	15,23 ± 5,84	16,45 ± 5,16	15,68 ± 3,16	14,77 ± 3,81	
	9,09 ± 6,42	10,82 ± 7,06	12,82 ± 6,65	12,00 ± 6,90	14,73 ± 5,47	14,73 ± 4,78	15,50 ± 4,24	14,23 ± 3,66	
	8,54 ± 6,82	10,23 ± 6,41	10,00 ± 5,36	11,68 ± 6,38	12,86 ± 5,72	13,64 ± 4,08	12,68 ± 5,05	12,45 ± 3,66	
	**I**^**a**^									
**Control**									
**N**^**a**^	**S**^**a**^								**T**^**a**^
	16,28 ± 2,95	16,06 ± 2,34	16,83 ± 2,12	16,61 ± 1,91	17,78 ± 2,16	17,22 ± 2,18	16,44 ± 2,62	16,67 ± 2,47	
	16,61 ± 2,25	17,61 ± 2,15	18,17 ± 1,54	18,44 ± 1,62	18,44 ± 1,62	18,22 ± 1,80	17,33 ± 1,68	18,00 ± 2,28	
	17,56 ± 2,12	18,83 ± 1,54	19,67 ± 0,77	19,56 ± 0,86	19,67 ± 0,77	19,67 ± 0,77	19,33 ± 1,68	18,67 ± 1,68	
	18,50 ± 1,38	19,56 ± 0,86	19,89 ± 0,47	19,67 ± 1,03	19,89 ± 0,47	19,89 ± 0,47	19,67 ± 0,77	19,67 ± 0,77	
	18,89 ± 1,57	19,89 ± 0,47	19,89 ± 0,47	19,89 ± 0,47	20,00 ± 0,00	20,00 ± 0,00	19,56 ± 0,86	19,56 ± 1,29	
	18,78 ± 1,40	19,44 ± 0,92	19,78 ± 0,65	20,00 ± 0,00	19,78 ± 0,65	19,78 ± 0,65	19,78 ±0,65	19,22 ± 1,70	
	17,67 ± 2,40	19,00 ± 1,24	19,61 ± 0,92	20,00 ± 0,00	20,00 ± 0,00	19,89 ± 0,47	19,78 ± 0,65	19.78 ± 0,94	
	17,39 ± 1,58	18,50 ± 1,62	19,00 ± 1,41	19,67 ± 1,03	19,56 ± 1,10	18,44 ± 4,20	19,33 ± 1,19	19,56 ± 0,86	
	**I**^**a**^									

^a ^S: superior; N: nasal; T: temporal; I: inferior

The most severe relative differential sensitivity deficits in ADOA were localized to the infero-nasal quadrant.

Mean ± standard deviation values of posterior pole retinal sensitivity measured by MP1 and Statistical Differences in ADOA patients and controls are shown in Table 5.

**Table 5:** Mean PP differential sensitivity values measured by MP1 comparing Autosomal-Dominant Optic Atrophy (ADOA) and Control Subjects.

Group	Retinal Sensitivity (dB)	Total
**ADOA**	**Mean**	12,86
	**SD**^**a**^	6,18
**Controls**	**Mean**	18,76
	**SD**^**a**^	1,87
	***P***^**b**^	.001

^a^**SD**: Standard Deviation

^b^***P***: Significant difference (*P*<0.05) in Anova test between normal and ADOA groups for each population

Furthermore, in the ADOA group 100 retinal areas with an absent response to maximal light stimulation (0 dB) were observed (7,8%), while no healthy subjects with absolute scotomas were identified.

A GCL thickness cut-off value of 23.5 micron corresponding to 0 dB sensitivity in the microperimetry map was identified in 74% of ADOA patients.

The mean values of BCEA and the mean values of the major and minor axis of the ellipses were significantly increased in the ADOA group (*P* = 0.001).

No correlation was found between BCVA and perimetric indices (Mean deviation, Pattern Standard Deviation, Visual Field Index) and average RNFL and/or GCL retinal thickness.

## Discussion

Previous studies carried out with TD-OCT have shown a significant reduction in average RNFL thickness in patients affected by ADOA [2,20,21,27,34,35]. These data have been confirmed in other studies, reporting significant thinning of the pRNFL in the infero-temporal quadrant using the Fast RNFL scan protocol of TD-OCT [2,36].

Ito et al. described thinning of the macular inner retina using TD-OCT manual segmentation, with preservation of the photoreceptor layer [35].

More recently, thickness of selected retinal layers has been measured using computer- assisted manual grading software analysis of the Spectralis SD-OCT macular scans [25]. RNFL and GCL thicknesses were significantly lower in ADOA patients than in controls, while there was no difference in IPL+INL+OPL and ONL+photoreceptor layer thicknesses [25,28]. Furthermore, a reduction of perifoveal RGC-IPL thickness was found in ADOA patients [27,37].

In this work, the Spectralis SD-OCT segmentation software was applied to the entire retinal posterior pole (and not only to the macular area) of ADOA patients.

Consequently, a greater proportion of the retina potentially affected by structural damage was successfully measured layer-by-layer using this new method. Repeatability and reproducibility data of the measurements of the different retinal layers at the retinal posterior pole obtained with the new Spectralis segmentation algorithm, have been published recently [31]. However, this automatic segmentation technique has not yet been used for measuring the entire Posterior pole thickness in previous studies on patients with ADOA.

Our results demonstrate a significant thinning of the inner layers at the retinal posterior pole, as measured by the new Spectralis automatic segmentation algorithm.

In particular, a significant reduction in thickness of the Retina, RNFL, GCL, IPL, INL and IRL was found in the retinal posterior pole when compared to the control group.

Measurement of the peripapillary RNFL thickness represents a clinically validated supporting parameter in the diagnosis and follow-up of various ocular diseases.

Our data demonstrated a thinning of the RNFL not only at the peripapillary level but also in the whole posterior pole of ADOA patients. Moreover, we found a strong correlation between PP RNFL and pRNFL thickness values. This finding suggests that PP RNFL, as obtained by Spectralis Automatic segmentation, could be a useful biomarker in identifying the neural loss in ADOA and in other retinal diseases. Furthermore, it implies the possible validity of Spectralis automated segmentation of the PP, as pRNFL encompasses all RGC axons.

However, alterations of the pRNFL thickness may also be caused by high myopia, tilted disc, papilledema and pseudopapilledema. Alternatively, ocular diseases other than ADOA (e.g. glaucoma, epiretinal membranes, macular edema) might influence Posterior Pole RNFL thickness.

Therefore, clear differentiation of the GCL and the RNFL at the posterior pole as separate entities, together with the measurement of their respective thickness as obtained by Spectralis automated segmentation, may be of particular interest in providing a more accurate and reliable structural evaluation of the eyes of ADOA patients even in the presence of comorbid ocular conditions.

Histopathologic changes in eyes obtained from postmortem specimens of two patients with ADOA have been reported. A decrease in the number of RGC and degenerative changes of residual ganglion cells mainly in the posterior pole were found; the number of papillomacular retinal nerve fibers was also decreased. As already reported in previous studies, this probably reflects a predilection of the pathologic process for the smallest fibers, most of which constitute the PMB [27,37]. Since OCT has become available, the fact that the RGC degenerate first in ADOA, with pRNFL loss developing secondarily, has been well established [14,15,19,20].

Our findings are congruent with these typical features of ADOA: as expected, in addition to GCL thinning, the PP RNFL thickness reduction was more consistent at the level of the PMB.

The value of the PMB thickness provided by the Spectralis confirmed the common pattern of retinal nerve fiber loss in these patients. Moreover, the RNFL thinning profile rendered the typical pattern of fiber loss strikingly evident in most of the patients’ eyes. Not only the PMB, but the whole temporal sector is flagged as “abnormal” on the Spectralis RNFL report, immediately resembling a dominant optic atrophy case.

Also, we found a slight but significant increase in OPL and ONL thickness in our ADOA patients.

A previous study reported significant outer retinal layer thickening (ONL including inner and outer photoreceptor segments) in 3 out of 58 ADOA patients with macular microcysts (MM) of the INL [38]. Two other previous studies reported the presence of MM of the INL of ADOA patients using an adaptive optics fundus camera and SD-OCT [28,39].

However, our patients did not display macular microcysts and carried different *OPA1* mutations as compared with patients from those studies; moreover, Spectralis automatic segmentation allowed us to measure ONL and OPL thickness separately. Thus, notwithstanding the validity of the hypothesis that the underlying retina must either expand or split to form MM as a consequence of the GCL and RNFL thinning and in the presence of a firm vitreo-retinal adhesion, the possibility that other mechanisms could contribute to these results is open to conjecture.

A possible explanation may be somewhat related to Müller cell functions. These cells span the entire retina, enveloping the photoreceptor cell bodies in a reticular network, and extending side processes to synapse in both plexiform layers. Müller cells contribute to regular neuronal function by clearing excitotoxins and glutamate, producing neurotrophic factors and aiding consistent processing of information at the neuronal level. Müller cell stimulation promotes retinal photoreceptor and neuron survival regardless of the underlying genetic or pathological defect leading to neuronal cell death. Virtually every disease of the retina is associated with reactive Müller cell gliosis, which supports the survival of retinal neurons [40].

In light of these facts, on analysis of the SD-OCT scans we discovered several hyperreflective dots in 4 ADOA patients at the level of the parafoveal ONL. It can therefore be hypothesized that degenerative ADOA processes could give rise to Müller cell activation and reactive gliosis resulting in hyperreflective microareas in this layer.

Although it would have been necessary to increase the sample size to reliably state that thickening of ONL and OPL was related to the compensatory behavior of Müller cells, it is conceivable that these cells, ubiquitous in the retina, may also contribute to the presence of the hyperreflective dots found in some of the SD-OCT scans mentioned above.

As expected, Retinal Pigment Epithelium and Outer Retinal Layers thickness values were not significantly different when compared to controls.

A previous study evaluating macular light sensitivity and fixation patterns using MP1 in patients with ADOA demonstrated unstable fixation and subnormal microperimetric sensitivity in the central and nasal macula where the ganglion cell deficit was most pronounced [26].

**Fig 5:**
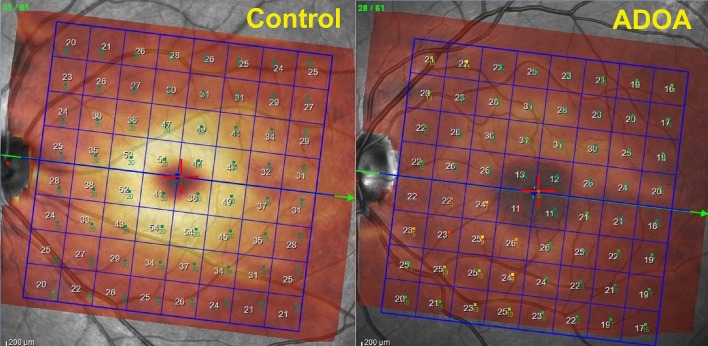
Combination map of SD-OCT GCL and MP1 PP map of a healthy subject vs ADOA patient.

Our results demonstrated a significant decrease of mean differential light sensitivity in the microperimetric Posterior Pole map of the ADOA group versus controls. Furthermore, most of the 100 MP locations with absolute scotoma (0 Db) in ADOA patients were in the infero-nasal quadrant, in direct correspondence with the most significant reductions in the Posterior Pole GCL thickness map (Fig 5). As expected, fixation stability measured by BCEA also proved to be significantly worse in our ADOA patients as compared to controls.

Our prospective cross-sectional study combined the assessment of different retinal layer thicknesses along with light sensitivity in patients with ADOA, using SD-OCT and MP.

The higher degree of correlation found between the thickness of specific retinal layers and differential fundus-perimetry light sensitivity values, as opposed to that measured between different retinal layers thicknesses and perimetric indices of standard automated perimetry, can be in part explained by the attempt to achieve congruence between structure and function pursued in this study. The development of an MP program that topographically traces the PP SD-OCT map has most likely enabled closer association between retinal volumes and the corresponding light sensitivity values.

Although the functional complexity of the visual apparatus prevents acquisition of a perfect point-to-point correlation between structural alterations and corresponding functional alterations in the visual field, the possibility of a closer topographic association between MP light sensitivity values and the volumes of the different retinal layers, permitted by "en face" segmentation of each layers, offers further interesting prospects. This simple, inexpensive technique, which involves superimposing a microperimetric map directly onto the OCT imaging, could improve the identification of functional impairment along with retinal alterations represented in a single map (Fig 2). For example, it would allow the identification of posterior pole GCL thickness values combined with corresponding sensitivity values to together on one map (Fig 5). These advantages may be mainly attributed to the developments made in the "Posterior Pole" MP program pattern. Both the SD-OCT and MP perform eye tracking during eye examination, guaranteeing topographical accuracy of the results as well as ensuring accuracy of the follow-up test by identifying specific tissue landmarks.

Other MP patterns may be developed and superimposed onto alternative SD-OCT maps.

However, the correspondence between the absence of photoreceptors, construed as the absence or destruction of the "internal segment/outer segment" in SD-OCT scans, consolidated by lack of sensitivity (0 dB) in the corresponding microperimetry values, may be immediately intuitive, whereas the correspondence between thickness values of the inner retina and light sensitivity values may not be equally so.

It is a fact that retinal thickness alone is not a good indicator of corresponding visual function integrity, while thinning of inner retinal layers is associated with a wide range of reduction of sensitivity values. Previous studies have shown that neurodegeneration leaving less than about 50 microns of parafoveal outer retinal thickness completely abolished light sensitivity in a few cases of patients with diseases of the inner retina [41].

In ADOA patients we identified a wide range of inner retinal thickness reductions associated with the appearance of a value equal to 0 dB in the microperimetry results.

Nevertheless, it has been possible to identify, if only for GCL thickness, a cut-off value of 23.5 microns, below which absolute scotomas appear in 75% of cases in the PP fundus perimetry map.

In the remaining layers, the identified cut-off becomes subject to lower sensitivity and specificity.

Utilization of the segmentation application of the Spectralis OCT employed in the present work permitted all retinal layers to be distinguished and measured separately. In this study, no manual correction of the automated segmentation algorithm was performed. A limitation of the present study is the cross-sectional design and its small sample size. Further studies are needed to examine whether manual corrections will improve the capability of the Spectralis OCT segmentation software to detect retinal alterations in patients with ADOA.

Optical coherence tomography and microperimeter MP1 measurements represent possible valid endpoints in the evaluation of the efficacy of new therapies in upcoming trials, although further studies are needed to further define the role of these techniques in the diagnosis of ADOA.
